# A 6-Week Badminton-Based Movement Intervention Enhances Fundamental Movement Skills and Physical Fitness in Saudi Boys and Girls

**DOI:** 10.3390/sports11070132

**Published:** 2023-07-07

**Authors:** Michael J. Duncan, Abdulrahman AlShabeb, Katie Fitton Davies, Nadia Alshahrani, Yazeed Almasoud

**Affiliations:** 1 Centre for Sport, Exercise and Life Sciences, Coventry University, Coventry CV1 5FB, UK; 2 Department of English, College of Languages and Translation, Imam Mohammed Ibn Saud Islamic University (IMSIU), Riyadh 11564, Saudi Arabia; amashebeeb@imamu.edu.sa; 3 Research Institute for Sport and Exercise Sciences, Liverpool John Moores University, Liverpool L3 2AJ, UK; k.fittondavies@ljmu.ac.uk; 4 Technical Programs, Applied College, Princess Nourah bint Abdulrahman University (PNU), Riyadh 11564, Saudi Arabia; 5 Saudi Badminton Federation, Riyadh 11564, Saudi Arabia; y.almasoud@badminton.sa

**Keywords:** physical education, motor skill, motor competence, test of gross motor development, object-control, strength, speed, power, cardiovascular endurance

## Abstract

This study examined the short (pre-post) and longer-term (post to 10 weeks post) effects of the Badminton World Federation (BWF) Shuttle Time program on fundamental movement skills (FMS) and physical fitness in Saudi boys and girls. Seventy-six children aged 9–11 years (44 boys, 32 girls; mean ± SD = 10.2 ± 0.9 years) undertook twice weekly Shuttle Time sessions for 6 weeks. Pre, post, and 10 weeks post, FMS was measured using the test of gross motor development 3 and physical fitness was measured via 10 m sprint speed, standing long jump, seated medicine ball throw, and a 6 min walk test (6MWT). There were significant improvements in FMS from pre to post (*p* = 0.0001) and post to 10-weeks post (*p* = 0.0001) for both boys and girls. Girls demonstrated a significantly greater magnitude of change in FMS. For 10 m sprint time, seated medicine ball throw, standing long jump, and 6MWT performance, both boys and girls significantly improved from pre-10 weeks post (all *p* = 0.001). The magnitude of change was greater pre to 10-weeks post for girls for the seated medicine ball throw, but was greater for boys for the standing long jump and 6MWT. In terms of practical applications, the BWF Shuttle Time program is feasible for administration in the Saudi context and is beneficial in developing FMS and fitness for Saudi children aged 8–12 years.

## 1. Introduction

Lack of physical activity (PA) is a critical health concern and remains the fourth leading risk factor for premature mortality, being responsible for 6% of death globally [1]. The benefits of PA are well documented [2], including lower risk of non-communicable diseases (NCDs) such as obesity, diabetes mellitus, coronary artery diseases, and hypertension, as well as having positive effects on, mental health, academic achievement, and quality of life. There is also robust evidence that PA habits developed during childhood track into adolescence and adulthood [3]. Consequently, the promotion of PA during childhood and adolescence is a critical public health concern. This is particularly the case in Saudi Arabia, where only 17.4% of the total Saudi adult population meets the WHO guidelines [4,5]. The Ministry of Health initiatives and Saudi’s Vision [6] agenda acknowledge the current challenges around physical inactivity locally and stress the importance of promoting PA through sport participation, particularly in children to improve the current and future health and prosperity of the Saudi population.

Over the past several decades, Saudi Arabia has witnessed enormous economic growth and prosperity, accompanied by technological transformation that has led to major negative changes in lifestyle [7]. In particular, physical inactivity and increases in sedentary behaviors have contributed significantly to an increase in lifestyle-related NCD’s placing a substantial health burden on the country [7]. There have been several robust cross-sectional studies undertaken in the last decade with Saudi children and youth, including the Arab teens and lifestyle study [8,9] and others [10]. Collectively, these aforementioned studies and the most recent review of physical inactivity in Saudi Arabia [7] conclude that the majority of Saudi children and youth are not active enough to meet the minimal weekly requirements of moderate to vigorous (PA) recommended by international guidelines. Furthermore, a review of the factors negatively affecting PA participation in Saudi children and youth concluded that the most influencing determinants appear to be age, obesity, low cardiorespiratory fitness, high television viewing, and poor physical education program [8]. There is, therefore, a need for sustained efforts to provide Saudi children with the tools to engage in physical activity to prevent future ill health. While physical education programs have been highlighted as one effective means to instill such habits in Saudi children [10], a lack of skills related to PA is a noted barrier to PA in Saudi adults [11,12] and school girls [7] in particular.

One major reason for the failure of initiatives to increase PA worldwide, and in Saudi Arabia particularly, has been a focus on children attaining 60-min of moderate to vigorous PA daily whilst assuming that children are “naturally” competent, confident, and motivated to undertake and engage in different forms of PA. However, considerable data demonstrate that children are not mastering their fundamental movement skills (FMS) to their expected age-related developmental capability [13,14,15,16,17,18]. To date, there are no data that provide information regarding the FMS of children in Saudi Arabia. FMS is widely recognized as an important determinant of PA and weight status in children [19,20], and mastery of FMS is purported to be essential for the development of more specialized movement patterns enabling youth to participate in organized and non-organized physical activities and sport over the life course [21,22,23]. FMS is globally defined as locomotor (e.g., running, jumping), object control (e.g., throwing, catching), and stability (e.g., balancing and twisting) movement categories [21,22], and, importantly, is not naturally acquired during the process of maturation [24]. To develop proficiency in FMS, there is a need to implement developmentally appropriate activities, specifically, teaching and learning activities (with feedback) during the provision of school-based physical education, alongside sufficient opportunities to practice for children and youth made available [25]. Interventions focusing on helping children develop FMS, usually through physical education or sport, have shown success in enabling children to be more physically active [25,26] and have a solid theoretical basis for longer term behavior change [23]. In line with conclusions drawn from systematic reviews and meta-analyses [27], there is a real need to develop effective strategies for children to develop their FMS to provide a solid foundation for future participation in sport, exercise, and PA. Moreover, although there are a considerable number of studies reporting on the FMS levels of children across the world, as yet, no data on this topic exist for children in Saudi Arabia. The current proposal seeks to address these issues by examining if a badminton-based FMS intervention is effective in promoting motor competence and physical fitness in Saudi boys and girls. Of note, in the context of Saudi Arabia, physical education has only been permitted for girls since 2017, with badminton being one recognized sport that girls are allowed to participate in. A key tenent of the Saudi Vision 2023 agenda [6] focuses on PE as an enabler for subsequent physical activity and health. There is to date no data reporting on effects of PE based intervention on Saudi girls in particular. This is a key gap and a need for research addressing this issue to understand the effect of any PE-based intervention on FMS, fitness, or other variables in Saudi girls in particular. Without this, the development of the PE curriculum and policy change promoting PE for girls may be less forthcoming.

The focus on a badminton-based intervention is a key facet of the current proposal as it is a sport where engagement has been shown to result in positive effects on physical fitness, blood pressure, and resting cardiovascular function in untrained adults [28] and reductions in body mass index in English children [29]. It is also a gender-neutral sport where both boys and girls can equally access participation, and as such the focus of this proposal directly aligns with the Kingdom of Saudi Arabia Vision 2030 objectives [6] related to increasing participation in sports and athletic activities. Badminton as a permitted sport for girls offers an opportunity to engage children in movement development through PE where the sport itself is multidimensional in terms of movement and can prepare children for participation in a wider range of sports than badminton alone, including other racquet and court sports [29,30]. The Badminton World Federation (BWF) Shuttle Time program [30] may be particularly useful as a vehicle to help Saudi children on a positive trajectory of health as badminton is a key activity for both boys and girls within the Saudi physical education curriculum. Moreover, the BWF Shuttle Time program has previously shown promise in improving FMS in English children [29]. The BWF Shuttle Time program was developed in 2012 to provide an effective means to develop FMS and badminton-related skills for children aged 5–15 years. Given the multi-dimensional movement demands inherent in badminton, the effective development of FMS is a key foundation for later performance in the sport, as well as other racquet sports. Implicit within the activities included in the Shuttle Time program are the embedding and development of FMS that, although badminton-related, likely also apply to a range of sports and physical activities. For example, developing competence in striking, jumping, and leaping, which are common skills in badminton, and developed as part of Shuttle Time, would facilitate future participation in sport and PA that employ those skills. In the aforementioned work by Duncan et al. [29], English children undertook one Shuttle Time lesson per week, in lieu of statutory PE for a 6-week period, with the authors reporting significant short-term improvements in FMS, physical self-efficacy, and reductions in unhealthy weight. In the Duncan et al. [29] study they established the efficacy of the BWF Shuttle Time program on children’s FMS compared to a control group who undertook their statutory PE lessons. Thus, the efficacy of the BWF Shuttle Time program is known. However, in the context of Saudi Arabia, where the opportunity to engage in different types of PE differs between boys and girls, the sport of badminton offers an important opportunity for girls to develop movement skills. With PE only being formally allowed for girls by the Saudi government in 2017, and with badminton being one of the sports girls are permitted to engage in, it is important to establish the effect of undertaking the BWF Shuttle Time program for girls, in comparison to boys. A first step needed before the intervention could be advocated wholesale is to examine the efficacy of the BWF Shuttle Time program in the Saudi Arabian educational context. This is due to physical education within the Kingdom of Saudi Arabia differing from other regions of the world. Therefore, the current study included two main aims: (1) to assess FMS and physical fitness in children in Saudi Arabia, and (2) to determine the effect of a twice-weekly, badminton-based intervention on short (pre-post) and longer-term (post to 10 weeks post) changes in FMS and physical fitness in Saudi boys and girls aged 8–12 years. We hypothesized that participation in the intervention program would result in improvements in FMS and fitness post-intervention.

## 2. Materials and Methods

### 2.1. Design

This study employed a repeated-measure intervention design where two classes of children from two schools, one boys’ school and one girls’ school, participated in the study. Given the aforementioned work by Duncan et al. [29] which established the efficacy of Shuttle Time compared to a control group, and the context of PE in Saudi Arabia in relation to boys and girls, we sought to compare the effect of the Shuttle Time intervention on boys and girls, defacto using boys as a form of control group, where having a matched control group of both sexes was not possible in the context which the intervention took place. The schools, which were specialist sports schools, were located in Riyadh, Saudi Arabia, in an affluent area of the community. All children undertook an assessment of FMS and physical fitness before and after undertaking the BWF Shuttle Time intervention. Both boys’ and girls’ groups undertook a structured, twice-weekly Shuttle Time program over a six-week period in place of one statutory physical education sessions and lasted 60 min with 50 min being spent as “time on task”. Boys and girls undertook the program separately and participation was congruent with prior work that has used the BWF Shuttle Time intervention with English school children [29]. Full guidance on how the Shuttle Time program was administered is presented in the BWF Shuttle Time teacher manual [30]. Prior to, immediately following, and 10 weeks post the intervention, participants in both schools were assessed on measures of FMS and physical fitness. The program was administered by PE teachers who had been trained in delivering the BWF Shuttle Time program by trained facilitators of the Saudi Badminton Federation, who are members of the BWF, the world governing body of the sport.

### 2.2. Participants

Seventy-six children aged 9–11 years (44 boys, 32 girls; mean ± SD = 10.2 ± 0.9 years) participated in this study following institutional ethics approval, written informed parental consent, and child assent. Height (cm) and body mass (kg) were assessed using a Seca Stadiometre and weighing scales (Seca Instruments, Hamburg, Germany) with the children barefoot and wearing the physical education kit (shorts and t-shirt for boys, trousers and t-shirt for girls). Mean ± SD of height and body mass were: 144 ± 9.1 cm and ± 42.6 13.4 kg, respectively. Body mass index (BMI) was determined at kg/m^2^ and mean ± SD of BMI was 20.5 ± 4.7 kg/m^2^, with 37.5% (*n* = 27) of the sample being overweight or obese based on IOTF cut-points [31]. Mean ± SD of anthropometric characteristics for boys and girls at pre-assessment are presented in Table 1. Inclusion criteria were being healthy with no musculoskeletal impairment, illness, or injury, during the intervention and including the 10-week post-assessment, not engaging in any other organized sport, other than school PE, during the intervention period.

### 2.3. Measures

Process and measurements of FMS and product measures of physical fitness were employed in the present study. Process-oriented movement skill assessment determines how the skill is performed [32], while product measures determine the outcome of the movement [33]. Using both process and product measures in this way, therefore, provides a holistic evaluation of FMS as has been recommended [33].

### 2.4. FMS

FMS was assessed using the test of gross motor development 3 (TGMD-3) [34]. The TGMD-3 is a well-validated process assessment of FMS and comprises 13 skills grouped into locomotor and object control subsets. In the current study, the following skills were assessed: run, hop, skip, gallop, jump, slide (Locomotor subset), two-hand strike, one-hand strike, dribble, overarm throw, underarm throw, kick, and catch (object control subset). Each skill comprises 3–5 components and an assessment of whether or not each component of the skill is performed is used to determine mastery of the skill [34]. Each skill was video recorded (Sony video camera, Sony, UK) and edited into single clips of individual skills. Quintic biomechanics analysis software v21 (Quintic Consultancy Ltd., Warwickshire, UK) was employed for this purpose alongside a process-oriented checklist. Scores from two trials were summed to obtain a raw score per skill. As per TGMD-3 guidelines [34], scores for skills were summed to create a total motor competence (scored 0–100) score in addition to scores of locomotor (0–46) and object control (0–54) competence. In all cases, assessment and scoring followed recommended guidelines of administration of the TGMD-3 [34]. The video clips of each skill were analyzed by two researchers with experience assessing children’s movement skills. Both raters had training in the assessment of children’s skill performances and comparison of their scoring to “gold standard” rating, following recommended procedures for scoring the TGMD-3 [3]. Training was considered complete when scores for the two trials differed by no more than one unit from the instructor’s score for each skill (>80% agreement) [35]. Inter- and intra-rater reliability analysis was performed for all the motor skills between the two researchers. Intraclass correlation coefficients for inter and intra-rater reliability were 0.895 (95% CI = 0.85–0.95) and 0.957 (95% CI = 0.92–0.97), respectively, demonstrating good reliability [36].

### 2.5. Physical Fitness

Four product measures of physical fitness, namely 10 m flying sprint time, standing long jump (SLJ), seated medicine ball (1 kg) throw (MBT), and 6-min walk test, were assessed. Procedures were congruent with those used previously [37] in evaluations of school-based interventions. A 10-meter sprint run was timed using Smart Speed gates (Fusion Sport, Coopers Plains, Australia). Two infra-red gates were set up 10 meters apart. The participant had a flying start (1 m) to ensure that sprint speed (Secs) was measured independently of the acceleration phase. Standing long jump (cm) was measured using a tape measure and following the procedures previously described [37]. The seated MBT was conducted, using a 1 kg medicine ball, as a measure of upper body strength following procedures reported by Davis et al. [38]. The seated MBT has been identified as a reliable and valid measure of upper body strength in children as young as 5 years old [38]. Children threw the medicine ball forward like a chest pass whilst being sat on the floor with legs extended. Three attempts were undertaken with the furthest distance thrown (cm) assessed using a tape measure. Children were instructed to throw the medicine ball with both arms and where a throw was executed with the use of only one arm, the trial was repeated. Reliability of the fitness tests employed in the present study in children of the ages taking part, with intraclass correlation coefficients of 0.9, 0.83, 0.94, and 0.85, has been reported previously [37,38].

### 2.6. Cardiorespiratory Endurance

The six-minute walk test (6MWT) was conducted as a measure of cardiorespiratory endurance [39]. Participants were instructed to walk as fast as possible (without running) on a flat sports hall surface between two markers, 30 m apart, for a six-minute period as per recommended guidelines [39]. The 6MWT is a practical, feasible, and well-established measure for assessing cardiorespiratory fitness in field-based settings [39]. Participants were instructed to walk as fast as possible (without running) for 6 min. After 5 min, the time left was advised to the participant. No other commands or verbal feedback was given. The number of laps completed was tallied for each participant and from this total distance covered calculated on test completion.

### 2.7. Shuttle Time Intervention

The present study employed a six-week version of the BWF Shuttle Time program [30]. No specific optimum duration of the Shuttle Time program is specified by the BWF and a six-week trial period was chosen as, pragmatically, it fits within a school half term, making it attractive for teachers for potential rollout in multiple schools. This decision was taken, congruent with studies examining the efficacy of school-based movement interventions [15,26], to have little disturbance on the school curriculum, to be time efficient, and to create a design that could be realistically integrated into the school curriculum. This process follows the only other published study employing the BWF Shuttle Time intervention which also employed a 6-week duration in 6–7-year-old children in England [29]. Delivery and focus of the intervention sessions followed the guidance provided by the BWF in their teacher’s manual [30]. Sessions were delivered by a teacher, a sports coach, and a researcher in each of the schools. Those delivering the sessions were the same sex as the pupils they taught. The Shuttle Time program was progressive, based on the exercises and activities specified by the BWF, and consisted of a warm-up section (10 min) and a main body section (approx. 40 min). The intervention focused on the development of the following: balance, coordination, underhand throwing, catching, striking, running, jumping, and correct use of a racquet (to grip and swing) [30].

The teachers delivering the intervention documented adherence to the program and compliance during the six-week period via registers. Any child who missed more than one session in the intervention period was not included in the final analysis. This resulted in four exclusions (all boys) from the final data set for analysis.

Table 2 outlines the content and schedule of the Shuttle Time program. Similar to other research using this approach with children [15,37,40], participants in the intervention groups also received skill-specific feedback on the quality of each movement during intervention sessions.

### 2.8. Statistical Analysis

A series of repeated-measure ANOVAs were used to examine any changes in dependent variables; FMS and physical fitness were assessed pre, post, and 10 weeks post the intervention period. Gender was used as a between-subject variable. In this way, we sought to assess any pre–post changes in dependent variables pre- and post-intervention and between sex groups. Where any differences were found, Bonferroni post hoc analysis was undertaken to determine where differences lay. Partial eta squared (*p*η^2^) was used as a measure of effect size and alpha level was set as *p* = 0.05 to indicate statistical significance. The Statistical Package for Social Sciences (SPSS version 28) was used for all analyses.

Recognizing that some children in the sample may have been starting their adolescent growth spurt, a posteriori, we reanalyzed the data using analysis of covariance (ANCOVA), controlling for maturity offset. The Moore et al. [41] prediction equation was used to determine maturity offset as a marker of biological maturation. This subsequent analysis enabled us to account for maturation by proxy. The mean ± SD of maturity offset was −2.68 ± 0.68 years, and there was no significant difference in maturity offset between boys and girls (*p* > 0.05, mean ± SD of maturity offset was −2.66 ± 0.77 years for girls and −2.71 ± 0.61 years for boys). The results of this analysis did not change the outcomes of the initial statistical analysis, and maturity offset was not significant as a covariate in any of the analyses (all *p* > 0.05). This analysis is not subsequently discussed any further.

## 3. Results

Mean ± SD and 95% confidence intervals for all variables, pre, post, and 10-weeks post-assessment for boys and girls are presented in Table 3.

### 3.1. FMS

**Figure 1:**
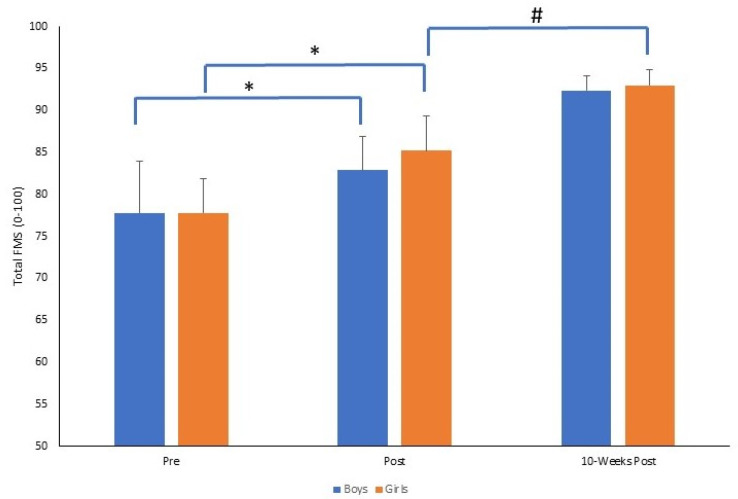
Mean ± SD of total FMS score (0–100) for boys and girls across time points (* *p* = 0.001, # *p* = 0.01).

For the total FMS score, there was a significant time by gender interaction (*F*_2,140_ = 3.372, *p* = 0.037, *p*η^2^ = 0.046). There were significant improvements from pre to post (*p* = 0.0001) and post to 10-weeks post (*p* = 0.0001) for both boys and girls. There was no significant differences between boys and girls at pre (*p* = 0.942) and at 10-weeks post (*p* = 0.172). However, immediately post-intervention, girls demonstrated significantly greater FMS than boys (*p* = 0.019), where the magnitude of change was greater pre to post for girls (Δ = 7.5) compared to boys (Δ = 5.1). The mean ± SD of the total FMS score for boys and girls across time points is presented in Figure 1.

**Figure 2:**
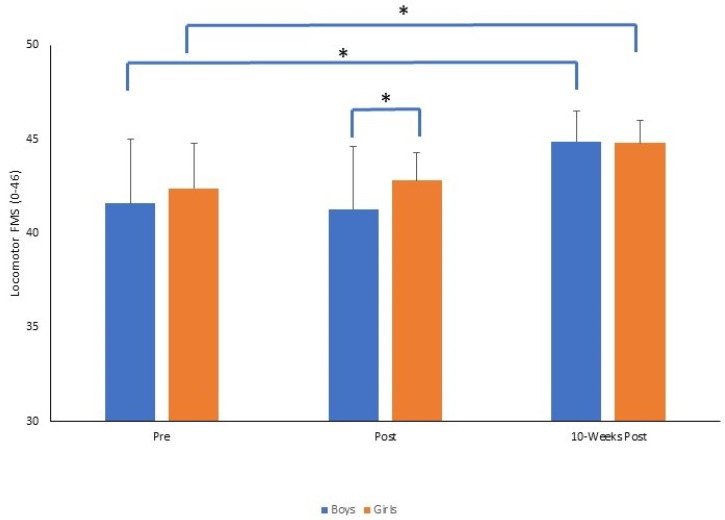
Mean ± SD of locomotor FMS score (0–46) for boys and girls across time points (* *p* = 0.001).

**Figure 3:**
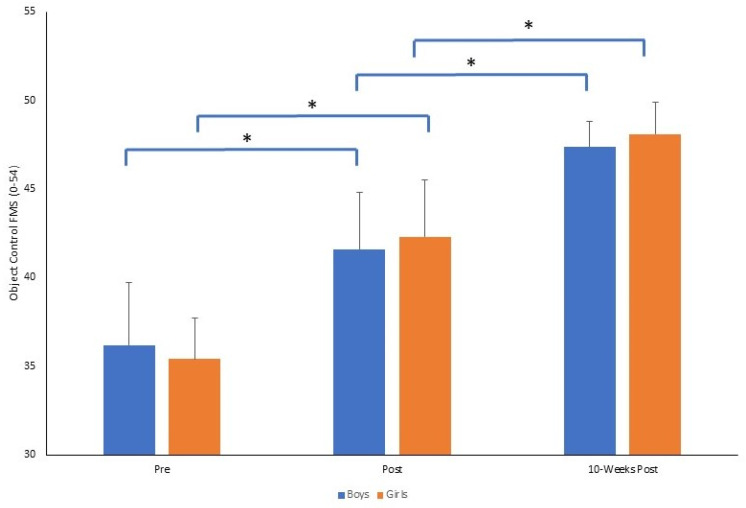
Mean ± SD of object control FMS score (0–54) for boys and girls across time points (* *p* = 0.001).

The results reported for total FMS were replicated when examining the TGMD subtest scores for locomotor (See Figure 2) and object control skills (See Figure 3). For locomotor skills, there was a significant time by gender interaction (*F*_2,140_ = 4.710, *p* = 0.01, *p*η^2^ = 0.063). There was no significant difference between boys and girls at pre (*p* = 0.267) and also at 10-weeks post (*p* = 0.388). However, immediately post-intervention, girls demonstrated significantly greater FMS than boys (*p* = 0.001), where the locomotor FMS had declined pre–post for boys but increased for girls. Conversely, from post to 10-weeks post, there was a greater magnitude of change in locomotor FMS for boys (Δ = 3.6) compared to girls (Δ = 2). For object control skills there was also a significant time by gender interaction (*F*_2,140_ = 4.170, *p* = 0.017, *p*η^2^ = 0.056). There were significant increases in object control FMS from pre to post for both groups (both *p* = 0.001) and from post to 10-weeks post. (both *p* = 0.001). There was no significant difference between boys and girls at pre (*p* = 0.299), but at post (*p* = 0.001) and 10-weeks post (*p* = 0.001) girls demonstrated significantly better object control skills than boys, where the magnitude of change was greater pre- to 10-weeks post for girls (Δ = 12.7) compared to boys (Δ = 11.1).

### 3.2. Physical Fitness

**Figure 4:**
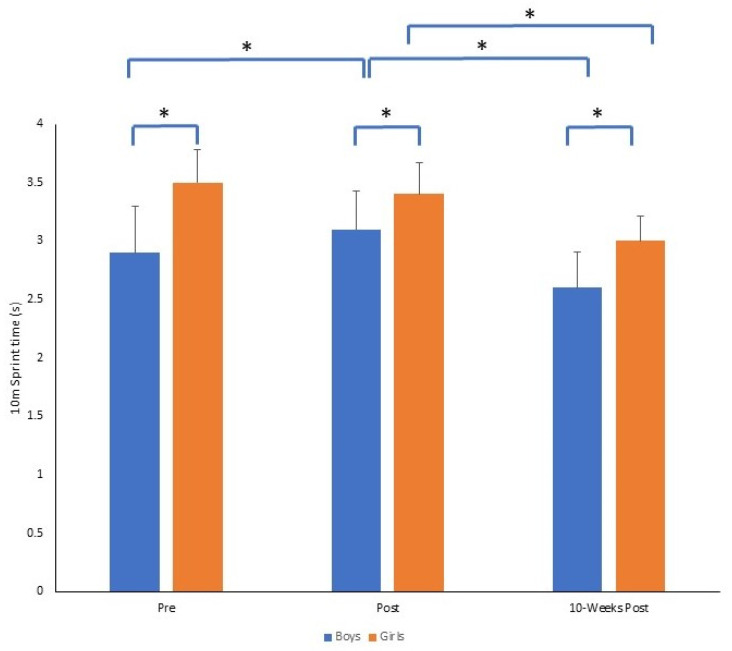
Mean ± SD of 10 m sprint time (s) for boys and girls across time points (* *p* = 0.001).

In regard to the 10 m sprint time, there was a significant time by gender interaction (*F*_2,140_ = 11.01, *p* = 0.001, *p*η^2^ = 0.136). At each time point, boys were significantly faster than girls (all *p* = 0.001). Boys also ran the 10 m distance in a significantly slower time from pre to post (*p* = 0.001) and then significantly faster from post to 10-weeks post (*p* = 0.001). Boys were significantly faster at 10-weeks post compared to pre (*p* = 0.001). For girls, there was no significant difference in 10 m sprint time from pre to post (*p* = 0.990), but girls were significantly faster at 10-weeks post-intervention compared to immediately post-intervention (*p* = 0.001). The mean ± SD of 10 m sprint time for boys and girls across time points is presented in Figure 4.

**Figure 5:**
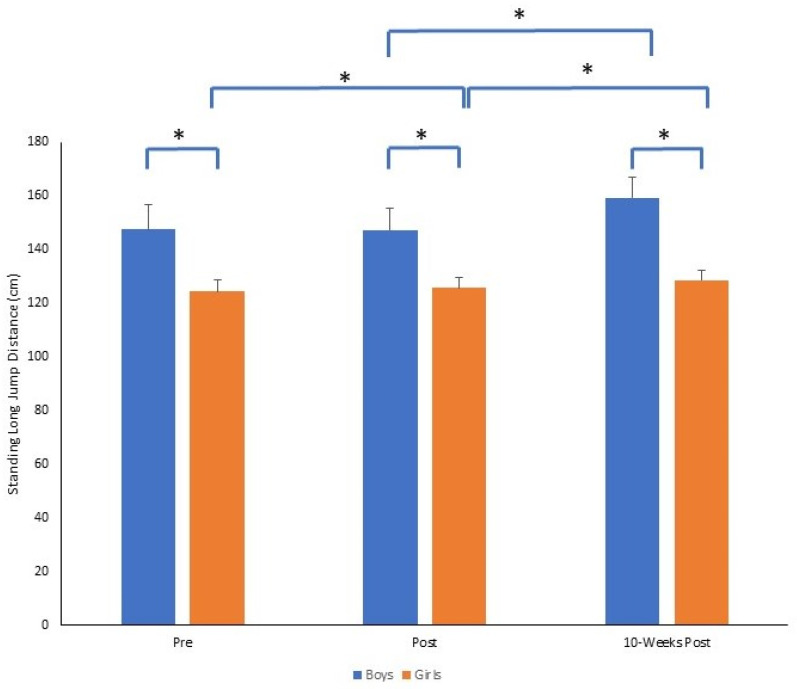
Mean ± SD of standing long jump distance (cm) for boys and girls across time points (* *p* = 0.001).

For standing long jump performance, there was also a significant time by gender interaction (*F*_2,140_ = 14.1, *p* = 0.001, *p*η^2^ = 0.168). At each time point, boys jumped significantly further than girls (all *p* = 0.001). Girls improved standing long jump performance from pre to post, and post to 10-weeks post (both *p* = 0.001), whereas boys did not improve pre to post (*p* = 0.367) but performed significantly better from post to 10-weeks post (*p* = 0.001). The magnitude of change was greater pre to 10-weeks post for boys (Δ = 11.7) compared to girls (Δ = 4.3). The mean ± SD of standing long jump distance (cm) for boys and girls across time points is presented in Figure 5.

**Figure 6:**
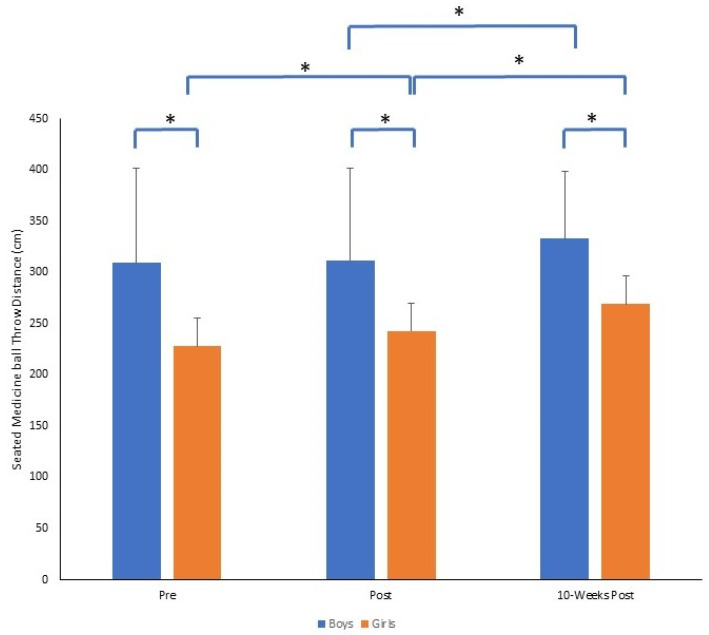
Mean ± SD of seated medicine ball throw distance (cm) for boys and girls across time points (* *p* = 0.001).

For seated medicine ball throw performance, there was also a significant time by gender interaction (*F*_2,140_ = 6.39, *p* = 0.002, *p*η^2^ = 0.084). At each time point, boys performed significantly better than girls (all *p* = 0.001). Girls improved medicine ball throw performance from pre to post, and post to 10-weeks post (both *p* = 0.001), whereas boys did not improve pre to post (*p* = 0.409) but performed significantly better from post to 10-weeks post (*p* = 0.001). However, the magnitude of change was greater pre to 10-weeks post for girls (Δ = 41.2) compared to boys (Δ = 23.6). The mean ± SD of seated medicine ball throw distance (cm) for boys and girls across time points is presented in Figure 6.

**Figure 7:**
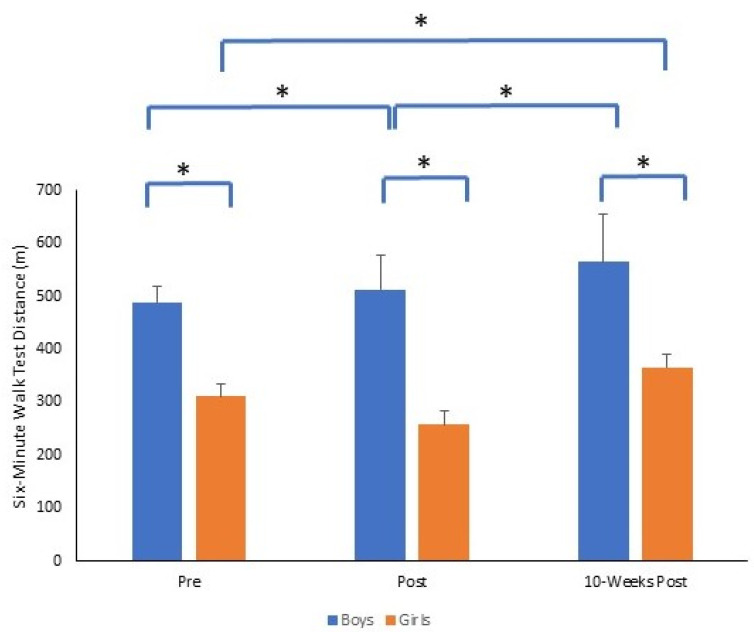
Mean ± SD of six-minute walk test distance (m) for boys and girls across time points (* *p* = 0.001).

Regarding the six-minute walk distance, similar to the other tests of physical fitness, there was also a significant time by gender interaction (*F*_2,140_ = 15.1, *p* = 0.001, *p*η^2^ = 0.177). At each time point, boys performed significantly better than girls (all *p* = 0.001). Six-minute walk distance significantly improved for boys from pre to post and from post to 10-weeks post (*p* = 0.001). For both boys and girls, the six-minute walk test distance was significantly higher at 10-weeks post, compared to pre for both boys and girls (both *p* = 0.001). The magnitude of change was greater pre to 10-weeks post for boys (Δ = 77) compared to girls (Δ = 55). Mean ± SD of six-minute walk test distance (m) for boys and girls across time points is presented in Figure 7.

## 4. Discussion

This study is the first to assess FMS in children within the Kingdom of Saudi Arabia and the first to examine the effect of the BWF Shuttle Time program on movement skills and physical fitness of boys and girls in Saudi Arabia. Hence, the results of the present study represent an original contribution to the literature regarding FMS development in children. We examined the short-term (pre–post intervention) and longer-term (post-10 weeks post intervention) effects to provide an indication of the longer-term retention of any change as a result of the program.

The present study supports the assertion that Shuttle Time enhances children’s FMS [29]. In the present study, scores for total FMS increased from pre to post, and at 10-weeks post for both boys and girls. There was a particular improvement in object control FMS specifically. This is key, as object control skills are considered more difficult for children to develop than locomotor skills and have been identified as a key barrier to engaging in physical activity. Of particular note in the present study, girls’ total FMS developed to a greater extent than that of boys. Such a finding is of practical importance because the lack of skills related to PA is a noted barrier to engaging in PA for Saudi Adults [11,12] and school girls [7] in particular. The development of FMS provides the building blocks for children to engage in different forms of PA and provide a positive trajectory for engagement in PA across the life course [23]. Likewise, the lack of quality physical education programs has been highlighted by prior work as a key determinant of the low levels of PA seen in Saudi children [8], whilst also being recommended as one effective means to instill PA habits in Saudi children [10]. The results of the present study demonstrate that the Shuttle Time program, undertaken in school Physical Education, can provide benefits to boys and girls in terms of FMS development, with a particular benefit for girls.

The results of the present study also suggest that engaging in the BWF Shuttle Time program resulted in sustained benefits to children’s physical fitness. Children in the present study undertook assessments of speed (via 10 m sprint speed), lower body muscular performance (via standing long jump), upper body muscular performance (via seated medicine ball throw), and cardiorespiratory fitness (via the six-minute walk test). For both boys and girls, performance in these tests improved. Boys improved to a greater extent than girls in the 10 m sprint speed, and standing long jump performance, whereas girls improved to a greater extent than boys in the seated medicine ball throw test. Low levels of fitness, particularly cardiorespiratory fitness, have also been identified as a key determinant of the low levels of PA seen in Saudi children [8]. Therefore, the improvement in physical fitness seen in the present study pre-post the Shuttle Time program should be considered a positive outcome of the present study.

Collectively, the results demonstrated in the present study offer positive evidence for the Kingdom of Saudi Arabia in relation to children’s PA and sport participation at grassroots levels. The results of the present study, despite being a pilot study in nature, highlight the feasibility of embedding badminton, and the BWF Shuttle Time program within school physical education, which could be further developed across the kingdom. Badminton is a gender-neutral sport where both boys and girls can equally participate. The results of the present study, therefore, align with and directly support the objectives of the Kingdom of Saudi Arabia Vision 2030 [6] in relation to increasing children’s participation in sports and athletic activities. It is also important to highlight that the findings of the present study are not only restricted to badminton. A key tenant of the Shuttle Time program is the development of competence in FMS that is developed through badminton but is applicable to a range of different sports and physical activities [30]. As engaging in the Shuttle Time program enhanced children’s FMS, theoretically, such enhancement would lead to a greater uptake of PA and sport participation, as increased movement competence (via FMS) leads to increased confidence to engage in sport and PA, with increased enjoyment and motivation to continue participation. This, in turn, creates a positive trajectory of health through FMS and PA [23]. This assertion would however need empirical testing in the context of Saudi Arabia.

To the authors’ knowledge, the results of the present study are the first to report on the effect of a school-based movement program on FMS and fitness of boys and girls in Saudi Arabia (or any of the Gulf countries). The present study suggests that school-based movement interventions enhance both fitness and FMS [15]. The present study would also support the prior work of Duncan et al. [29], in English children, younger than those examined in the present study, which suggested the BWF Shuttle Time program could enhance FMS if undertaken within school physical education in the English PE curriculum. The notable increases in object control skills observed in the current study are of particular note. Meta-analytical data [26] have suggested that interventions focusing on object control skills may be of greater benefit to overall FMS development that those focusing more on locomotor skills. The nature of the Shuttle Time program is such that object control skills are a key feature. The activities within the intervention itself comprise directed learning tasks with specific coaching cues and manipulation of task constraints [42], such as the use of balloons instead of shuttlecocks or the removal of badminton nets during the early stages of the intervention, which are considered the most important factor of Newell’s [42] constraints-led approach in developing movement skills [43]. For example, balloons slow down the trajectory so that children have more time to respond while maintaining information coupling. This more easily transfers learning to when progressing to shuttlecocks and also necessitates broader development of object control skills for striking than would be the case for shuttlecocks alone. Likewise, some of the activities in the Shuttle Time program focus on throwing specifically without needing a shuttlecock to be returned by a partner. This facilitates the development of shoulder, elbow, and wrist pivots needed for the FMS of throwing and striking. Moreover, providing opportunities for more success during the earlier stages of learning (through task simplification) promotes feelings of competence and therefore continued engagement. We speculate this approach is the primary mechanism responsible for the improvement in FMS reported in the present study.

Understanding if the BWF Shuttle Time program is effective in enhancing FMS and fitness is a needed first step in establishing the evidence base for an intervention that, although used in practice in different countries [30], has never been employed in the context of Saudi Arabia, and where scientific evidence for its effect has yet to be established. Where an existing program is used but without empirical support for its efficacy, a key factor in refining intervention efficacy is determining if the “one size fits all” approach as originally proposed in the Shuttle Time program is effective for children in different cultures and contexts. The current study provides this evidence in the context of the Kingdom of Saudi Arabia. Although the format and content of PE differ in Saudi Arabia to other countries, particularly for girls, and as a result, the results of the present study may not be as generalizable to other areas of the world, the results of the present study may be practically relevant for other gulf countries where the PE curricula are similar.

### Limitations and Direction for Future Research

The current study is not without limitations. We are conscious that there was no control group in the current study. We recognize this as a limitation, however, given the efficacy of the BWF Shuttle Time program has been established against a control group previously [29] and given the context of the current study where girls have only been permitted to engage in PE in Saudi Arabia since 2017, comparing the effect of the same program on boys and girls is important, and in this sense, the boys’ group acts as a control where both boys and girls undertook the same program. This is a key first step in establishing the efficacy of Shuttle Time in the context of physical education within Saudi Arabia with a particular emphasis on the experience of girls. However, considering the positive findings of the present study, future work using a randomized controlled trial design would be welcome. Likewise, the current study provides evidence of positive change in FMS and fitness as a consequence of undertaking the Shuttle Time program over a six-week period. It is possible that undertaking the program might also influence other variables such as mental well-being and PA, amongst others. We were not able to assess habitual PA during the intervention, nor were we able to assess body composition. Both would have been useful data to have collected. Similarly, we did not assess maturity status. Instead, we used the Moore et al. [41] prediction equation and used this as a covariate when reanalyzing the data to control for maturation. This reanalysis made no difference to the results of the statistical analysis. All participants were pre-pubertal at the time of assessment. Future studies would be welcome to examine if there is a change in PA, or mental well-being as a consequence of undertaking the BWF Shuttle Time intervention alongside the changes we documented related to FMS. We are also cognizant that the data presented here report the effect of a six-week Shuttle Time program undertaken twice per week, in lieu of statutory physical education. A six-week period was undertaken as prior work [15] demonstrated this duration of motor competence intervention, including the only other published study using Shuttle Time [29], can be effective and, importantly, fits within the demand of a crowded school curriculum. The Shuttle Time intervention was also administered following the recommended guidance for teachers as given by the Badminton World Federation [11]. The pedagogic approach of the intervention is primarily based on directed learning with specific cues being provided by teachers/coaches to facilitate movement. The intervention was administered by movement-trained professionals (physical education teachers and coaches) and intervention fidelity was monitored using time on task. This confirmed that the children were physically engaged in each activity for approximately 45 min of each 50 min intervention session. A process evaluation of the intervention was not possible in the present study but would be useful in future work. Finally, the participants in the present study were recruited via convenience sampling from two schools that are in relatively affluent areas of Riyadh and are specialist sports schools. Consequently, the basic motor abilities of the participants may have been better than for the general population of children in the Kingdom of Saudi Arabia. The results of the present study would suggest, however, that as Shuttle Time improved FMS and physical fitness in this participant group, it is likely it may have a greater impact in groups who are not attending specialist sports schools. We would therefore suggest that the current study demonstrates evidence of promise for the Shuttle Time intervention as a program to enhance FMS and fitness in children in Saudi Arabia. The results of the current study could inform policy or curriculum development in Saudi Arabia, particularly for girls, by reinforcing the benefits of badminton-based movement intervention through PE or contributing to efforts aimed at promoting physical activity and sports participation among children, via the development of FMS through PE based intervention which can support engagement in PA as children and thereafter [23]. The current study should not be overstated but we would argue the results presented here suggest that if rolled out on a larger scale, Shuttle Time would result in successful impacts on movement skill development in children in Saudi Arabia.

## 5. Conclusions

To conclude, both FMS scores and physical fitness improved as a result of a six-week BWF Shuttle Time program with a greater magnitude of change in FMS observed for girls compared to boys. The current study suggests that the BWF Shuttle Time program is beneficial in developing FMS and physical fitness for children in Saudi Arabia and could be advocated as an effective strategy for schools to employ which will set children on a positive trajectory of movement development for PA and health benefit.

## Figures and Tables

**Table 1:** Anthropometric characteristics (mean ± SD) per group at pre-assessment.

	Boys (*n* = 40)	Girls (*n* = 32)	*p*-Value
Age (y)	10.2 ± 0.8	10.1 ± 1.0	0.74
Height (cm)	142.6 ± 9.3	146.5 ± 8.5	0.07
Body Mass (kg)	42.5 ± 15.1	42.6 ± 11.2	0.96
Waist Circumference (cm)	64.7 ± 10.7	65.7 ± 13.4	0.37

**Table 2:** Structure of the six-week Shuttle Time program.

Week 1	Week 2	Week 3	Week 4	Week 5	Week 6
Warm-Up (10-min)					
Balance Exercises	Balance Exercises	Balance Exercises	Balance Exercises	Dynamic Balance	Dynamic Balance
Mobility Exercises #1,2 and 4	Mobility Exercises #1,2 and 4	Mobility Exercises #1,2 and 4	Mobility Exercises #1,2 and 4	Mobility Exercises #1,2 and 4	Mobility Exercises #1,2 and 4
Having a Lunge	Having a Lunge	Having a Lunge	Having a Lunge	Having a Lunge	Having a Lunge
	Balance and Throw	Balance and Throw	Balance and Throw	Balance and Throw	Balance and Throw
Main Body (40-min)					
Balloon tap	Balloon tap relay (1) With hand (2) With Racquet	Mirror Chase with Throw and Catch	Mirror Chase with Throw and Catch	Mirror Chase with Throw and Catch	Mirror Chase with Throw and Catch
Balloon tap relay	Mirror Chase with Throw and Catch	Grip Change with Shuttle	Grip Change with Shuttle	Shuttle Chase	Shuttle Chase
Mirror Chase	Grip Change with Shuttle	Balance the Racquet	Throwing Game 1	Forehand and Backhand Lift Merry go Round	Forehand and Backhand Lift Merry go Round
Mirror Chase with Throw and Catch	Balance the Racquet	Throwing Game 1	Backhand Short Serve	Backhand Short Serve	Backhand Short Serve
Balancing Shuttles	Keep your Court Free	Chase and Hit (1) Forehand (2) Backhand	Chase and Hit (1) Forehand (2) Backhand	Flat Play	Flat Play
	Balancing Shuttles	Balancing Shuttles	Balancing Shuttles	Balancing Shuttles	Balancing Shuttles

Please note: The full BWF Shuttle Time program, including descriptions and videos of all the exercises, can be obtained from: https://shuttletime.bwfbadminton.com (accessed on 20 March 2023). Mobility exercises in the BWF Shuttle Time Peach are numbered #1–4, please see the shuttle time website for further detail regarding what each mobility exercise comprises.

**Table 3:** Mean ± SD and 95% confidence intervals for all variables, pre, post, and 10-weeks post-assessment for boys and girls.

Variables	Sex	Pre-Intervention [95%CI]	Post-Intervention [95%CI]	10-Weeks-Post-Intervention [95%CI]	ANOVA *p*-Value		
					Time	Sex	Sex × Time
Total FMS (0–100)	Boys	77.8 ± 6.1 [75.9–79.8]	82.9 ± 4.0 [81.6–84.2]	92.4 ± 1.7 [91.8–92.9]	0.0001	0.233	0.037
	Girls	77.8 ± 4.1 [76.2–79.2]	85.2 ± 4.1 [83.7–86.7]	93.0 ± 1.8 [92.3–93.6]			
Locomotor Skills (0–46)	Boys	41.6 ± 3.4 [40.5–42.7]	41.3 ± 3.3 [40.7–41.8]	44.9 ± 1.6 [44.7–45.2]	0.0001	0.047	0.01
	Girls	42.4 ± 2.4 [41.5–43.3]	42.8 ± 1.5 [42.3–43.4]	44.8 ± 1.2 [44.4–45.3]			
Object Control Skills (0–54)	Boys	36.2 ± 3.5 [41.3–43.2]	41.6 ± 3.2 [40.5–42.6]	47.4 ± 1.4 [46.9–57.9]	0.0001	0.739	0.017
	Girls	35.4 ± 2.3 [34.5–36.2]	42.3 ± 3.2 [41.1–43.5]	48.1 ± 1.8 [47.6–48.5]			
10-m Sprint Time (s)	Boys	2.9 ± 0.40 [2.8–3.1]	3.2 ± 0.33 [3.1–3.3]	2.6 ± 0.31 [2.5–2.7]	0.001	0.001	0.001
	Girls	3.5 ± 0.28 [3.4–3.6]	3.4 ± 0.27 [3.3–3.5]	3.0 ± 0.21 [2.9–3.1]			
Standing Long Jump (cm)	Boys	147.6 ± 9.1 [140.7–154.6]	147.3 ± 8.2 [140.6–154.0]	159.3 ± 7.6 [152.7–165.8]	0.001	0.001	0.001
	Girls	124.3 ± 4.6 [116.5–132.1]	125.7 ± 3.9 [118.2–133.2]	128.6 ± 3.7 [121.3–133.2]			
1 kg Seated Medicine Ball Throw (cm)	Boys	309.3 ± 92.2 [286.8–331.7]	311.9 ± 89.7 [290.1–333.8]	332.9 ± 66.2 [311.1–354.7]	0.001	0.002	0.001
	Girls	227.8 ± 27.8 [202.7–253.0]	242.7 ± 27.2 [218.3–267.3]	269.0 ± 27.3 [244.7–293.4]			
6-Min Walk Distance (m)	Boys	488 ± 30.8 [470.5–505.5]	512.5 ± 64.3 [494.9–530.1]	565.0 ± 89.2 [547.7–582.3]	0.001	0.001	0.001
	Girls	310.6 ± 24.8 [291.1–330.2]	357.5 ± 16.7 [337.8–377.1]	365.6 ± 13.6 [346.3–384.9]			

## Data Availability

Data are available from the first author upon reasonable request.
